# Curcumin as Therapeutic Modulator of Impaired Antioxidant Defense System: Implications for Oxidative Stress-Associated Reproductive Dysfunction

**DOI:** 10.3390/biology14070750

**Published:** 2025-06-23

**Authors:** Tuba Latif Virk, Qi Liu, Yuguo Yuan, Xianyu Xu, Fenglei Chen

**Affiliations:** 1 College of Veterinary Medicine, Yangzhou University, Yangzhou 225009, China; tuba.virk2@gmail.com (T.L.V.); qiliu@yzu.edu.cn (Q.L.); yyg9776430@163.com (Y.Y.); 2 Jiangsu Co-Innovation Center for Prevention and Control of Important Animal Infectious Diseases and Zoonoses, Yangzhou 225009, China; 3 Joint International Research Laboratory of Agriculture and Agri-Product Safety of the Ministry of Education of China, Yangzhou University, Yangzhou 225009, China

**Keywords:** curcumin, antioxidants, oxidative stress, follicle growth, female infertility

## Abstract

One of the critical challenges in assisted reproductive technology (ART) is the inadequacy of effective regulation of reactive oxygen species. Simultaneously, the endogenous antioxidant defense system plays a significant role in combating oxidative stress across various physiological stages of embryonic development. However, these intrinsic defense systems alone are insufficient as they rely on exogenous antioxidants that interact synergistically to enhance and sustain antioxidant capacity. Considering the principal role of antioxidants in mitigating oxidative stress in oocyte growth, identifying reliable and non-toxic antioxidants is an essential prerequisite for effective therapeutic applications. Thus, owing to the need to explore exogenous antioxidants, we attempted to summarize and analyze the literature data defining the potential use of curcumin in mitigating oxidative stress to promote oocyte maturation through *in vivo* and *in vitro* model studies. Recent studies demonstrated the protective role of curcumin against oxidative stress and the inflammatory response, primarily through the upregulation of key antioxidant enzymes (including SOD, CAT and GPx), a reduction in oxidative stress markers (e.g., ROS, MDA) and by suppressing the pro-inflammatory signaling pathways (such as NF-kB, JAK/STAT) while activating the NRF2/HO-1 pathway to further enhance the cellular antioxidant defense. Advancing curcumin as a therapeutic agent necessitates a thorough understanding of curcumin’s molecular mechanisms and targeted pharmacological effectiveness to treat female infertility, and despite the progress in enhancing curcumin’s bioavailability, the optimal dosing strategies still need to be defined. Future studies are required to develop strategies to augment antioxidant defense mechanisms (modeling *in vivo* and *in vitro* studies) using curcumin with a specific emphasis on curcumin’s role in improving mitochondrial activity. This approach is expected to represent a significant advancement in the field of medicine, offering novel therapeutic possibilities.

## Simple Summary

The ovary, being a metabolically active organ, is particularly sensitive to fluctuations in oxidative stress. Under physiological conditions, reactive oxygen species (ROS) play important signaling roles in ovarian functions. However, oxidative stress becomes detrimental only when ROS levels exceed the threshold level and lead to cellular damage, disrupted oocyte quality and impaired embryo development. An equilibrium between ROS and antioxidant enzymes in the ovary is essential to ensure the protection of growing oocytes and embryos. There is a rising need to explore for non-toxic, natural antioxidants to mitigate oxidative stress and simultaneously strengthen the endogenous antioxidant defense systems. The reduction in oxidative stress markers and enhancement of antioxidant capacity appear to be closely associated with curcumin’s direct free radical-scavenging ability, as well as its capacity to stimulate the activity of endogenous antioxidant enzymes. Therefore, we propose the potential of curcumin to improve reproductive efficiency while preserving cellular integrity and physiological relevance based on previous studies’ findings. This study offers valuable theoretical insights to support the use of curcumin in practical applications in enhancing antioxidant defenses and mitigating oxidative stress, thereby contributing to the maintenance of redox homeostasis within the ovary.

## 1. Introduction

Successful reproduction is a fundamental process to preserve the continuation of the life cycle, whereas 50% infertility has been reported by epidemiological studies over the past sixty years. The ovary is the major organ in the female reproductive system, as it facilitates the maturation and release of oocytes for potential fertilization. In addition, it also acts as a primary site for female steroid hormone synthesis. Through a complex interplay of both hormonal and non-hormonal signals, a series of processes (folliculogenesis, oogenesis and steroidogenesis) are regulated in a fully coordinated manner [1]. A precise equilibrium between reactive oxygen species (ROS) and antioxidants is crucial to regulate signaling mechanisms and biological pathways, thereby ensuring proper functioning of the female reproductive system [2]. However, oocytes and granulosa cells undergo oxidative damage due to an imbalance between ROS levels and antioxidant defenses, consequently leading to poor oocyte quality [3].

ROS can influence follicular development, oocyte maturation, ovulation, fertilization, embryo implantation and the early stages of embryonic development [4]. Remarkably, at low concentrations, in various physiological reproductive processes, ROS function as essential signaling molecule [5]. However, elevated ROS levels can lead to cellular damage by targeting organelles and DNA, disrupting enzymatic activities and inducing apoptosis [6]. In long-term *in vitro* culture systems, the optimum growth and development of follicles is attained under control conditions which significantly differ from their natural environment [7]. And subsequently, excessive ROS accumulations can occur due to supra physiological oxygen levels (up to 20%) [8]. There are various other factors contributing to oxidative stress *in vitro*, including temperature variations, large volumes of culture media, excessive light exposure, fluctuations in medium osmolality, high glucose concentrations, static nature of culture systems and inadequate antioxidant combinations [9]. Therefore, to mitigate oxidative stress during prolonged *in vitro* follicle culture in both humans and domestic animals, it is crucial to control the culture conditions intensively and implement an optimized combination of antioxidants at each stage of follicular development [10].

**Figure 1:**
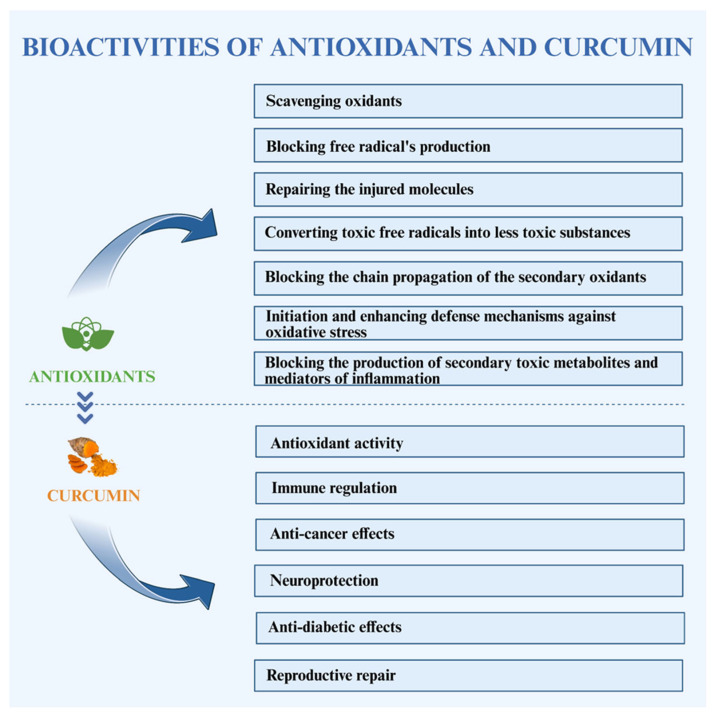
Overview of bioactivities of antioxidants and the associated health benefits of curcumin.

In recent years, the utilization of natural antioxidants has garnered considerable attention in scientific research. Bioactive compounds derived from plant sources have been studied extensively to mitigate oxidative damage by neutralizing free radicals and enhancing the endogenous antioxidant defense mechanism [11]. Among the diverse range of herbal therapies with an old history, turmeric (*Curcuma longa*) a plant from the Zingiberaceae family, has been extensively recognized for its medicinal properties and is often referred to as the “queen of medicinal plants” [12]. Traditionally, it has been widely incorporated into dietary and therapeutic applications, particularly into managing reproductive disorders (POCs and endometriosis) caused by ROS for disease prevention and health promotion [13]. Curcumin, a bioactive polyphenolic compound extracted from *Curcuma longa*, has been an integral component of Indian and Chinese herbal medicine for centuries [14]. Curcumin exhibits significant antioxidant potential, which is primarily attributed to the presence of key functional moieties (including -OCH_3_, -OH and C=C) within the molecular framework. As a standard phenolic antioxidant, curcumin exerts free radical scavenging activity by donating hydrogen atoms *via* phenolic groups, thereby neutralizing ROS and preventing oxidative damage [15]. Free radicals (ROS and RNS) are highly unstable species characterized by the presence of one or more unpaired electrons in their outermost orbitals. This instability stems from their inability to achieve a stable octet configuration. ROS and RNS exhibit characteristics such as the generation of reactive radicals, a short lifespan and the ability to cause destructive damage to various cellular contents [16]. ROS is continuously generated during normal physiological processes and trigger lipid membrane peroxidation, resulting in the accumulation of lipid peroxides [17]. Another significant destructive effect of ROS occurs in mitochondria, responsible for producing ATP through oxidative phosphorylation *via* an electron transport chain (ETC), and ROS can result in mitochondrial dysfunction [18]. Previous studies have reported curcumin’s pharmacological activities (Figure 1) including anti-inflammatory, antioxidant, anti-toxic, anti-apoptotic, anti-diabetic and immunomodulatory properties. *In vitro* assays and animal model experiments reported curcumin as a promising agent to mitigate the potential causes of female infertility and regulating female reproductive health [19,20,21,22,23]. Furthermore, several studies have demonstrated the stimulatory effects of curcumin on ovarian functions to minimize cell apoptosis while promoting cell proliferation and steroidogenesis [24]. Curcumin fortifies the endogenous antioxidant defense mechanism by upregulating the activities of antioxidant enzymes (SOD, GPx and CAT). Similarly, it mitigates the ROS generation by inhibiting the activity of pro-oxidant enzymes (such as xanthine hydrogenase/oxidase and lipoxygenase/cyclooxygenase) while maintaining cellular redox balance [25]. However, the Biopharmaceutics Classification System (BCS) has classified curcumin as a class IV compound due to its poor solubility and low permeability. These phytochemical limitations significantly limit curcumin’s oral bioavailability for absorption and systemic distribution [26]. However, modifying the structure of phenolic compounds has been shown to improve curcumin’s chemical and oxidative stability [27].

This review paper presents three critical aspects of curcumin’s role in reproductive medicine: (1) the challenges of curcumin’s bioavailability and stability under *in vivo* and *in vitro* conditions with potential solutions to enhance its effectiveness; (2) the safety and therapeutic potential of curcumin supplementation in assisted reproductive technology (ART) and its impact on fertility outcomes; and (3) a comprehensive analysis of recent studies highlighting curcumin’s potential in mitigating oxidative stress and improving ovarian function.

## 2. Reactive Oxygen Species (ROS)

Reactive oxygen species (ROS) are highly reactive and transient molecules with strong electrophilic characteristics and comprise both free radical (O_2_^−^) and non-radical species (such as H_2_O_2_). The increased concentration of O_2_^−^ and H_2_O_2_ induces oxidative stress [28]. Oxidative stress is a key concept underpinning the study of redox biology and its applications in medicine, first introduced in 1985 [29]. In biological terms, oxidative stress occurs when intracellular antioxidants are declined, or ROS accumulate excessively. It is frequently mentioned that ROS are involved in over 100 diseases including heart problems, kidney diseases, diabetes, infertility and cancer [14]. However, a direct measurement of the ROS level is not possible due to the extremely short half-life and alternations in biomarkers of ROS (such as malondialdehyde (MDA), superoxide dismutase capacity (SOD), total antioxidant capacity (TAC), catalase (CAT) and glutathione peroxidase (GPx)) which are commonly observed [30]. Across a significant number of cases, female infertility is associated with molecular damage to the ovary (oocytes or related tissues), primarily resulting from oxidative stress which interferes with the normal physiological processes of oocyte and other mammalian cells, thereby impairing reproductive function. ROS are also closely associated with the aging process and development of various reproductive diseases (POCS and endometriosis) [31]. Throughout the reproductive lifespan, fertility is broadly linked to the ovarian reserve (residual quantity of oocytes in ovaries); however, reproductive success is also contingent upon the quality of oocytes [32]. The developmental rate is an indicator to assess oocyte quality, as it directly reflects the efficiency of embryo production during culture [33]. ROS adversely affect oocyte competency and embryo development, leading to a decline in cleavage rates and overall developmental potential [34]. To alleviate oxidative stress during embryo culture, efforts have been made to supplement the culture media with antioxidants.

## 3. Need for Antioxidants

**Figure 2:**
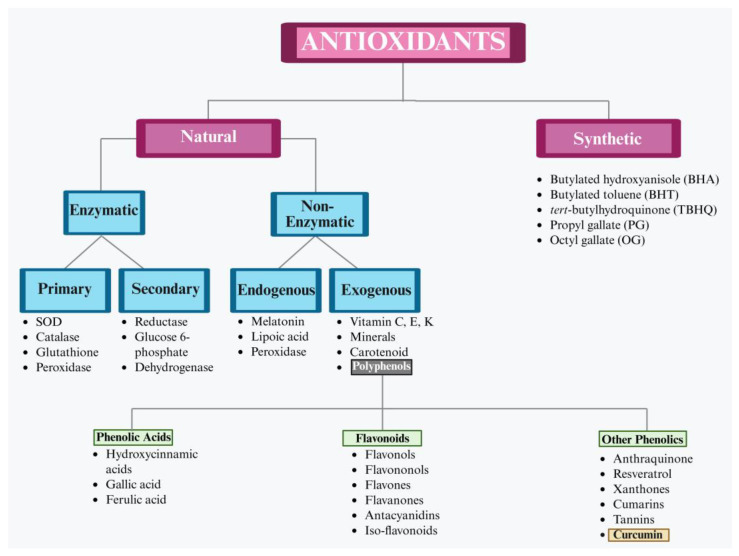
Classification of antioxidants based on their origin and mode of action, demonstrating curcumin as naturally occurring non-enzymatic exogenous antioxidant.

An antioxidant is a molecule capable of preventing the oxidation of macromolecules and primarily functioning to halt oxidative chain reactions by neutralizing free radicals [35]. Antioxidants are classified into two categories: (1) natural/biological and (2) synthetic [36]. Natural antioxidants are predominantly derived from plant sources such as fruits, vegetables, herbs and spices which are rich in bioactive compounds (including vitamins, phenolics, carotenoids and trace elements) [37], while synthetic antioxidants are chemically produced compounds like butylated hydroxyanisole and hydroxytoluene (Figure 2) [38].

A reduction in antioxidant capacity due to the insufficient presence of enzymatic and non-enzymatic antioxidants makes cells more vulnerable to oxidative stress. Generally, redox homeostasis is maintained through a precise balance between low ROS and the activation of the cellular defense mechanism [39]. In mammals, female reproductive performance is regulated by intricate mechanisms within ovarian tissues; therefore, maintaining cellular homeostasis (through a balance between ROS and the cellular antioxidant machinery) is crucial for proper oocyte development [40]. A combination of endogenous and exogenous antioxidants synergistically maintains the cellular integrity of the female reproductive microenvironment against ROS [41].

The relatively simple composition of embryo culture media lacks many antioxidant molecules typically present in the female reproductive system. Studies have shown that oocytes are subjected to three overlapping integral challenges in the process of development *via* the following: (1) maintaining dormancy in a state of low activity; (2) ensuring the reliable storage and transmission of critical biological information (including DNA, RNA, proteins and other organelles), which is essential for the survival of species; and (3) minimizing cellular damage and efficiently managing the accumulation of reactive oxygen species (ROS), thereby preserving cellular integrity and functionality throughout the development. Conversely, antioxidants play a significant role in animals’ defense mechanisms against ROS (Figure 1). These molecules operate three primary mechanisms: (1) directly scavenging ROS; (2) repairing damaged cellular components; and (3) deactivating ROS to mitigate their harmful impact on cellular structure and functions [42].

## 4. Curcumin

Several studies utilize antioxidants as a strategy to combat oxidative stress for successful oocyte development. A study showed significant improvement in the mitochondrial activity and blastocyst development in aged female mice supplemented with antioxidants [43]. However, at a large scale, numerous formulated antioxidant drugs are commercially available, while the predominant concern regards their toxic effects. This has led to a rising interest in the exploration of naturally derived antioxidants [44]. Natural antioxidants are primarily derived from plants (including edible vegetables, fruits, spices and herbs) which are abundant in vitamins, phenolics, carotenoids and micro-elements [45]. The United States Food and Drug Administration (USFDA) has declared curcumin as “generally regarded as safe” for both humans and animals. Moreover, extensive research has also demonstrated that curcumin possesses a diverse array of pharmacological properties with a favorable safety profile [46]. Additionally, over the past five decades, inclusive clinical studies have evaluated the safety, efficacy and pharmacokinetics of curcumin, endorsing a deep understanding of its therapeutic potential and biological mechanisms [47].

Curcumin is a polyphenol, has nearly a two-century-old history and was first isolated in 1815 by Vogel and Pelletier as a “yellow coloring constituent” from the rhizomes of *C. longa*, marking the discovery of this bioactive compound [48]. The term “polyphenol” or “phenolic” refers to a compound that contains an aromatic ring with one or more hydroxyl groups [44], and these compounds are recognized as one of the most abundant classes of phytochemicals known for their health-promoting roles in various biological functions [49]. *C. longa* is the principal source of curcumin with an average percentage of 2–5% of turmeric and consequently yielding an approximately 3% curcumin extract. However, curcumin concentrations can vary considerably among different specimens of the same species, while among all curcumin-producing species (including curcumin zedoaria, curcumin mangga, curcumin aromatic, curcumin phaeocaulis and curcumin xanthorrhiza), *C. longa* is the most widely cultivated species due to commercial applications [50]. Curcumin’s antibacterial properties were first documented by Schraufstatter, published in Nature in 1949, and this discovery triggered the scientific interest of researchers to further explore its pharmacological potential across different scientific disciplines. Cumulatively, comprehensive data persist, formalizing over 13,000 scientific publications on curcumin’s wide-ranging applications in medicine and food technology [51]. Additionally, the natural metabolites in curcumin have been shown to counteract oxidative stress and metabolic disorders [52]. Notably, phenols exhibit intrinsic antioxidant properties and can protect biomolecules (such as nucleic acids, proteins, poly-unsaturated lipids and sugars) from oxidative damage by intervening in free radical-induced reactions [53]. Evidence shows that curcumin possesses several pharmacological properties to fight against various diseases [45]. In recent years, curcumin’s potential role in reproductive medicine has gained traction, especially in addressing conditions such as PCOS, premature ovarian failure and oxidative stress-induced infertility [54].

## 5. Curcumin’s Antioxidant Mechanism: Scavenging Free Radicals and ROS Modulation at the Cellular Level

**Figure 3:**
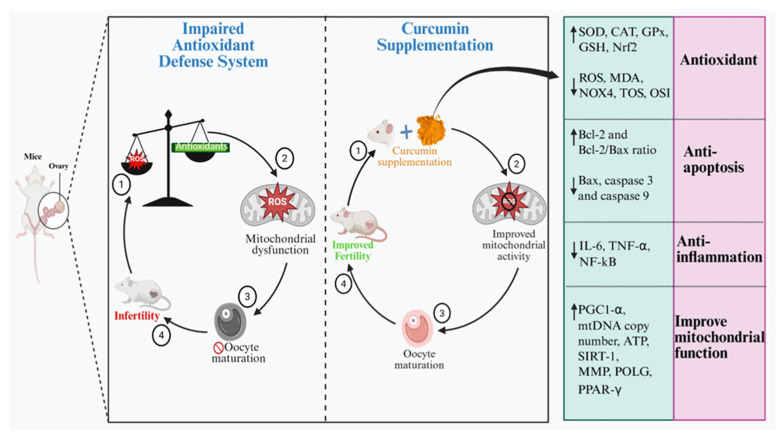
Schematic representation of two contrasting cycles: the impaired antioxidant defense mechanism leading to cellular damage and infertility and the effects of curcumin in attenuating ROS levels through multiple signaling pathways (↑ represents an increase; ↓ represents a decrease).

Curcumin can protect reproductive cells against oxidative stress and inflammatory damage (Figure 3) through its anti-inflammatory, anti-apoptotic and antioxidant properties [55]. Curcumin is found to exhibit significant antioxidant activity by efficiently interrupting the chain reactions involved in free radicals’ production. Curcumin’s capacity to scavenge hydrogen peroxide is notably superior when compared (at an equivalent concentration of 20 mM) to commercially available antioxidants such as butylated hydroxyanisole (BHA), vitamin E and butylated hydroxytoluene (BHT) [56]. Curcumin showed significant ability to attenuate lipid peroxidation and DNA damage against ROS due to potent antioxidant properties [57]. Granulosa cells (GCs), being the primary somatic cells within mammalian ovarian follicles, are integral for follicular development and reproductive functions. Granulosa cells play significant roles in steroidogenesis and the facilitation of ovulation [58]. Granulosa cells build a connection with growing oocytes, contributing to their structure, maintaining a shared microenvironment while minimizing ROS-induced damage by synthesizing antioxidants [59].

However, an overabundance of ROS within the follicular environment triggers apoptotic pathways; thereby, oocyte and granulosa cells become prone to damage. The reduction in oxidative stress markers and improved antioxidant capacity attribute to curcumin’s dual mechanism of action, strengthening the endogenous defense system against oxidative damage. The reason behind this regulatory effect is the direct free radical-scavenging properties of curcumin and its ability to upregulate antioxidant enzyme activity [60]. To ensure appropriate cell signaling, radical-scavenging enzymes regulate intracellular ROS levels, maintaining a threshold required for physiological functions. The primary mechanism underlying the antioxidative function of antioxidant enzymes is initiated by SOD which catalyzes the dismutation of O_2_^−^ into H_2_O_2_ [61]. Subsequently, a coordinated enzymatic system (CAT, GPx, GR and GST) facilitates the conversion of H_2_O_2_ and other reactive peroxides into water (H_2_O) and molecular oxygen (O_2_) through a multistep detoxification process. This cascade effectively mitigates oxidative stress and maintains redox balance [62].

Curcumin exhibits a highly efficient reactivity with superoxide radicals (comparable to those of established lipid-soluble antioxidants), and this interaction not only facilitates the neutralization of superoxide but also promotes catalytic degradation, collectively functioning as a superoxide dismutase (SOD) mimetic, effectively mitigating oxidative stress and preserving cellular integrity [63]. The α, β- unsaturated-β-diketo moiety of curcumin takes part in nucleophilic addition reactions (known as Michael addition reactions) where the unsaturated ketone acts as an electrophilic acceptor. This reaction involves nucleophilic donors (such as -OH, -SH, -she groups) that form covalent bonds with electrophilic carbonyl carbon. This mechanism is significant to understand the biological chemistry and reactivity of curcumin in cellular environments [64]. Several studies have proved that curcumin can directly scavenge ROS and reactive nitrogen species (RNS) and efficiently eliminates superoxide anions and hydrogen peroxide, thereby mitigating oxidative damage [65].

Furthermore, studies have shown that curcumin exerts beneficial effects on female reproductive health by promoting ovarian functions and treating the associated reproductive disorders [66]. However, increased levels of antioxidants have shown detrimental effects on various body organs possibly through reductive stress [67]. Likewise, the ameliorative effect of curcumin is concentration dependent, and curcumin’s high concentrations may exert toxic effects, potentially leading to cell death [68]. In addition, exceeded levels of antioxidants can induce excessive mitochondrial ROS production leading to cytotoxicity disrupting the glutathione redox balance [69]. However, current studies lack consensus on the precise concentration required to achieve consistent results, so further research is required to define evidence-based dosage guidelines, considering factors such as an individual’s physiological response to maximize its pharmacological efficacy in reproductive medicine.

## 6. Bioavailability of Curcumin

The fraction of the compound that is absorbed into the bloodstream, distributed through the systemic circulation, exerts its post-metabolic biological effects and subsequently is eliminated is called bioavailability, whereas the proportion of bioactive compounds released from the matrix during digestion and their availability for absorption is called bio-accessibility [70]. Curcumin has pharmacological properties; however, curcumin also poses significant challenges owing to its low bioavailability, unstable chemical structure, rapid metabolic degradation and limited adsorption in the body, and these factors hinder curcumin’s effectiveness in therapeutic applications [71]. Despite these significant limitations, extensive research continues, aiming to improve the bioavailability for therapeutic applications. These challenges have driven the development of innovative strategies aimed at overcoming the limitations of curcumin’s original native form. Various curcumin formulations have been designed to improve its permeability, bioavailability, systemic circulation and half-life while ensuring its stability against metabolic degradation [72]. Curcumin derivatives have emerged as promising alternatives to natural curcumin, offering improved bioavailability, metabolic stability and disease-targeting potential. These modified curcumin analogs demonstrate targeted activity against particular conditions (such as cancer, inflammation and neurodegenerative disorders) with more precise and effective treatment strategies [73]. Recent advancements in adjuvant research (including quercetin, piperine and genistein) and some other formulations (including liposomal encapsulation, nanoparticle-based delivery systems and the incorporation of absorption enhancers) have demonstrated significant improvements in curcumin’s bioavailability [74]. Liposomes are vesicular carriers (composed of one or more phospholipid bilayers) capable of delivering therapeutic agents directly to the target cells [75]. Studies demonstrate that hybrid liposomal formulations enhance curcumin bioavailability more effectively than conventional liposomes [76]. Nanostructured encapsulation strategies have further optimized curcumin’s therapeutic efficacy by improving its aqueous solubility, extending its half-life and enabling sustained drug release, thereby addressing its key pharmacokinetic limitations [77]. Nanoparticle-based curcumin formulations showed 69.78 times increased bioavailability compared to free curcumin [78]. Nano-curcumin *via* SPIONs reduced Bax, caspase-3 levels and increased Bcl-2 levels in the granulosa cells of mice, thereby promoting normal folliculogenesis [79]. This effect is largely attributed to the properties of nanoparticle delivery systems, which protect encapsulated drugs from premature degradation, enabling controlled and sustained release while facilitating targeted delivery to specific tissues or cells. Raja et al. (2021) [80] reported that curcumin-loaded (chitosan-based) nanoparticles effectively restored the ovarian morphology and function in POCS rats. Similarly, another study demonstrated that curcumin–selenium nanoparticles (CurSeNPs) significantly mitigated doxorubicin-induced ovarian toxicity in rats by decreasing the VEGF gene expression and improving the follicular development. Their findings highlight the antioxidant and protective effects of CurSeNPs on the ovarian structure and function [81].

## 7. The Fate of Curcumin in Cell Culture Systems: Stability, Uptake and Activity

To replicate the physiological processes of the female reproductive system, *in vitro* studies provide a highly controlled system for evaluating the fundamental aspects of reproductive biology [82]. Several follicle culture systems have been developed, advancing our understanding of reproductive mechanisms, showing clinical applications in preserving reproductive potential in endangered species and treating female infertility [83]. This method is also useful to conduct *in vitro* assays for assessing the potential reproductive risks associated with various supplements and drugs [84]. Moreover, the development of 3D culture techniques allows more precise replication of ovarian follicles’ distinct microenvironment and complex cell–cell interactions, ensuring the accurate representation of the follicular dynamics [85]. Although this culture system offers numerous advantages, debate continues over the most effective strategies for its establishment, focusing on the antioxidants used, along with their characteristics, permeability, toxicity and the ease and high success rate with which they can be manipulated and handled during follicle culture and harvest.

**Figure 4:**
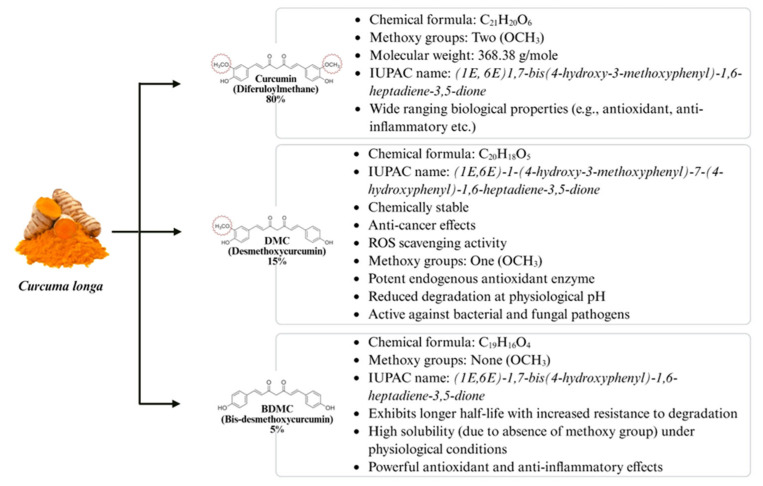
Inclusive profile of *Curcuma longa*, illustrating curcumin and the other two curcuminoids (DMC and BDMC) along with their distinguishing characteristics [87,88,89].

According to PubMed, over 3000 review articles have been published in recent years, demonstrating the effectiveness of natural antioxidants in preventing diseases associated with oxidative stress (Figure 4). Consequently, antioxidants have increasingly been integrated as co-adjuvants in conventional therapies aimed at combating oxidative stress [86]. However, the effectiveness of exogenous antioxidants mitigating oxidative damage remains a subject of debate, with a question mark over the chemical reactivity of exogenous antioxidants under *in vitro* conditions. Recent comprehensive reviews have addressed several key issues, including (1) the antioxidant effects of natural extracts sourced from various plant spices; (2) the mechanism underlying antioxidant activity; and (3) the preventive role of antioxidants in a range of diseases.

Numerous *in vivo* and *in vitro* studies have revealed the diverse beneficial biological activities associated with curcumin [90]. The chemical instability of curcumin is often disregarded when interpreting results from *in vitro* studies, although curcumin degrades rapidly at physiological pH, which slows in the presence of serum or cultured cells [91]. Furthermore, the color of curcumin exhibits pH-dependent variations, appearing bright yellow within a pH range of 2.5–7.0. In contrast, at alkaline pH levels exceeding 7, it undergoes structural transitions in a dark red coloration [92]. Currently, numerous curcumin formulations have been developed through scientific research with improved stability, though not all are commercially available. *C. longa* is a curcuminoid mixture (Figure 4) (75% curcumin, 15% desmethoxycurcumin (DMC) and 5% bis-desmethoxycurcumin (BDMC)) [93] which collectively enhances curcumin’s bioactivity, particularly antioxidant and antifungal properties [94]. It is worth mentioning that the two active curcuminoids (DMC and BDMC) in *C. longa* are natural and not synthetic as they possess structural similarity to curcumin and show improved stability and bioavailability [95]. However, distinguishing curcumin’s effects from its metabolites is challenging because the presence of DMC and BDMC may induce alteration in the degradation rate of curcumin, influencing the overall behavior in experiments [96]. However, curcumin’s degradation rate tends to decrease when it interacts with albumins, liposomes, lipid-based carriers, cucurbituril, cyclodextrins, surfactants, polymers and various macromolecules and micro-heterogenous systems [97]. Protein complexation has emerged as a promising approach to enhance the bioavailability and solubility of curcumin in aqueous environments, thereby improving pharmacokinetics and optimizing the antioxidant potential of curcumin [98]. Recently, protein-based carriers have been extensively explored in the field of biomedicine [99], including casein [100] and bovine serum albumin (BSA) [101]. Despite these significant advancements, further clinical trials are required to fully elucidate the biological relevance of curcumin’s breakdown products and their relative potential therapeutic role.

## 8. ROS in Ovarian Physiology and Reproductive Regulation

The female reproductive system, with its complex and multifaced nature, illustrates the vast scope of influence of ROS in regulating various physiological processes (folliculogenesis, oocyte maturation and fertilization) demonstrating the integral involvement of ROS in reproductive functions [102]. Oxidative stress leads to cell apoptosis, causing damage to DNA and other key organelles (including mitochondria and endoplasmic reticulum) and this damage triggers a cascade of molecular events that activate apoptotic pathways, leading to programmed cell death (Figure 3) [103]. Due to oocyte apoptosis, the number of gametes available for ovulation reduces while granulosa cells disrupt the supply to both oocyte and surrounding cells, making undesirable changes to the ovarian cellular microenvironment and impairing follicular function and development [104,105,106].

In the process of oocyte maturation, it has been evaluated that 60 ng/oocyte of ROS is required to maintain the oocyte in diplotene arrest [107]. However, a slight increase to 80 ng/oocyte of ROS can trigger the resumption of meiosis in critical developmental stages [108]. ROS are produced in significant amounts by vascular endothelial cells and macrophages when, during ovulation, a surge in luteinizing hormone (LH) triggers oocyte release and alongside neovascularization takes place, and this ROS production is essential for stimulating oocyte maturation and follicle rapture [109]. However, excessive ROS production can lead to cytotoxicity, disrupting the function of granulosa cells, impairing their ability to synthesize key steroid hormones, which can ultimately affect the oocyte quality. This mechanism collectively signifies the necessity of oxidants and antioxidants to provide protection to the growing oocytes and granulosa cells against oxidative damage [110,111].

## 9. Protective Role of Antioxidants in Counteracting ROS

Many oxidative stress measurements reported in the literature are classified as biomarkers (by the National Institute of Health) which are quantitative parameters of biological processes [112]. ROS are produced through various cellular metabolic processes of oocyte development and primarily as byproducts of ATP synthesis during mitochondrial respiration [113]. Endogenous antioxidant enzymes (SOD, GPx, CAT) function to neutralize free radicals at cellular and tissue levels. However, when superoxide production exceeds this defense capacity, ROS accumulate, leading to oxidative stress [114]. This mechanism signifies the delicate balance between ROS and enzymatic antioxidants which is necessary for the regulation of reproductive events [115]. Curcumin as a non-enzymatic exogenous antioxidant has been shown to be involved in influencing enzymatic activities related to ROS generation, nitric oxide synthesis, glutathione metabolism and phosphorylated Tau. The antioxidant activity of curcumin has been found to be approximately 10 times higher than that of vitamin E [116].

Extensive research over the past two decades has been devoted to understanding the mechanism underlying curcumin’s free radical scavenging activity to further elucidate curcumin’s properties to combat oxidative stress (Table 1). An animal-based experimental study showed the potent antioxidant properties of curcumin against oxidative stress, and these findings were further corroborated by another scientific study which reported that dietary turmeric supplementation significantly enhanced ovarian folliculogenesis, steroidogenesis and fertility outcomes [117]. In another study, different antioxidants were tested against oxidative stress in mice ovarian tissues, with curcumin showing significant results to reduce ROS levels [118].

## 10. Modulatory Effects of Curcumin in Improving the Ovarian Function

**Figure 5:**
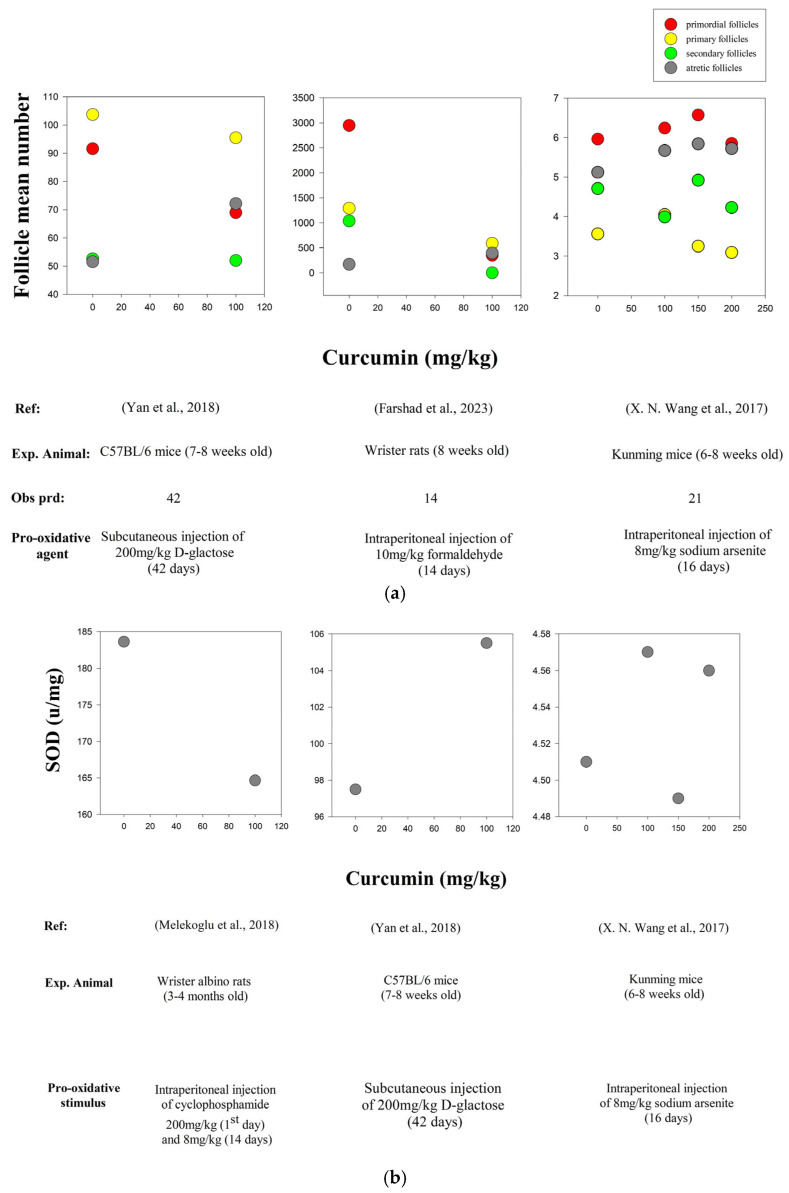

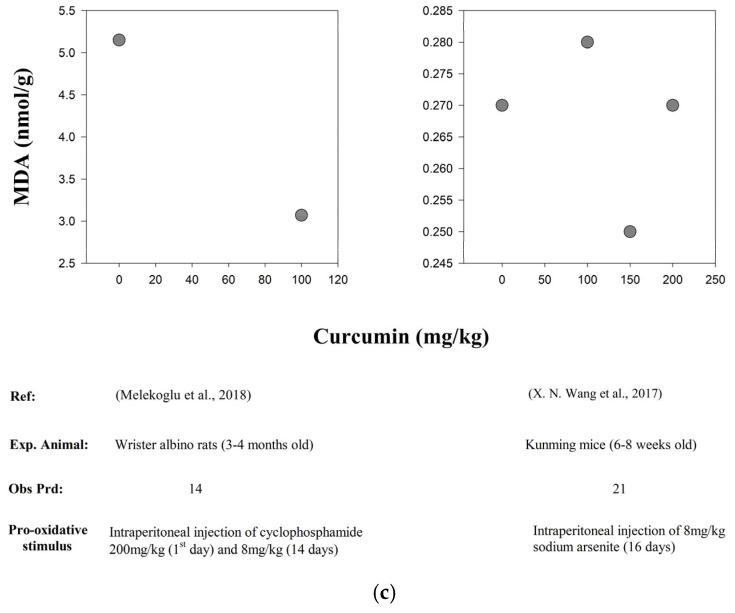
(**a**) Effects of different curcumin concentrations on ovarian follicle development under oxidative stress conditions [105,106,107]. (**b**) Curcumin-mediated enhancement of endogenous antioxidant enzyme activity under oxidative stress conditions [105,107,111]. (**c**) Curcumin-mediated cellular oxidative balance under oxidative stress conditions [107,111].

The mammalian ovary is a highly dynamic organ in the female reproductive system, where follicular growth serves as a physiological mechanism that regulates the selection and fate of growing follicles [122]. Curcumin modulates multiple intracellular signaling pathways, including PI3K/AKT/mTOR, JAK/STAT/ERK/MAPK, Wnt/β-catenin, NF-kB and p53. At the molecular level, curcumin influences key regulatory networks by modulating the expression of apoptotic markers (e.g., Bcl-2 and caspases) and enhances the enzymatic activity of endogenous antioxidants (e.g., SOD, CAT, GPx). These combined actions contribute to a reduction in oxidative stress while downregulating pro-apoptotic signaling and thereby reinforcing cell survival mechanisms [123]. Through the regulatory influence on transcription factors such as Nrf2 and NF-kb, curcumin initiates the upregulation of oxidative defense pathways, thereby strengthening cellular resilience against oxidative stress [124]. Lv et al. (2021) [125] provided compelling evidence that curcumin exerts a protective effect on the ovarian reserve by modulating the PTEN-AKT-FOXO3a pathway. Using single-oocyte gene expression analysis and *in vitro*/*in vivo* ovarian models, the study showed that curcumin reduces primordial atresia while maintaining follicle quiescence and improving ovarian reserve markers (AMH, FSH, E2) in aging mice. A study found that curcumin promoted the transition of follicles in mice from primary to secondary (*in vitro*) and curcumin-treated follicles developed into antral follicles when transplanted (*in vivo*). A study reported that curcumin enhanced the antioxidant defense mechanism by neutralizing free radicals, reducing the levels of oxidative stress markers (ROS, MDA) (Figure 5c) and by increasing the activity of endogenous antioxidants (SOD and CAT) (Figure 5b). A reduction in oxidative stress improved the follicle reserve (Figure 5a) and the histological profile of ovarian tissues. Cell survival increased and tissue repair was prompted with the downregulation of pro-apoptotic factors (Bax, cleaved caspase-3) and upregulation of anti-apoptotic proteins (Bcl-2) [126]. Y. Zhao et al. [127] reported that curcumin enhanced the granulosa cell proliferation, reduced cell apoptosis and activated the PI3K signaling pathway while suppressing the mTORC1 signaling pathway. Studies have revealed that curcumin promotes cell proliferation by activating the p38 MAPK pathway while upregulating anti-apoptotic regulators such as Bcl-2 and Akt signaling pathways [128]. Additionally, the role of MPAK in mediating curcumin’s effects has been observed in healthy ovarian cells [129]. Accompanied by another study, curcumin regulates multiple signaling pathways (including cellular homeostasis, oxidative stress and inflammatory pathways) by modulating the key molecules, p38 MAPK, NF-kB, Nrf2, β-catenin, COX-2, TNF-α, FOXO3 and VEGF [130]. Ahamed et al. (2022) [131] found that curcumin effectively mitigates copper oxide nanoparticle-induced toxicity in human placental cells by reducing oxidative stress, restoring antioxidant activity and regulating apoptosis-related gene expression. Table 1 summarizes the studies investigating curcumin supplementation with a particular focus on the modulated biological pathways involved in ovarian function (including experimental approaches and the key findings from *in vivo* and *in vitro* animal models).

## 11. Conclusions

The optimal regulation of reactive oxygen species (ROS) is crucial for maintaining cellular homeostasis in growing follicles. Living organisms rely on an intricate antioxidant system to maintain equilibrium between oxidative stress and antioxidant defense systems; thus, enhanced intracellular ROS breakdown and adequate antioxidant bioavailability is essential. When antioxidants are compromised, or the ROS level exceeds a threshold level, oxidative stress dysregulates redox reactions. *In vitro*, ROS are generated in oocytes as mitochondria utilize oxygen for energy, leading to excessive ROS accumulation, oxidative stress and potentially compromising oocyte quality. Notably, dietary antioxidants exhibit significant wide-ranging bioactivities, particularly in maintaining redox homeostasis. Curcumin is an antioxidant, capable of modulating the levels of reactive oxygen and nitrogen species, influencing multiple redox sensitive signaling pathways. However, curcumin presents a challenge in identifying whether the antioxidant-mediated effects are particularly due to curcumin or also can be attributed to the other curcuminoids (DMC and BDMC) present in *C. longa*. A key challenge is strengthening the mitochondrial antioxidant defenses, which depends on precise cellular targeting. The pharmacological benefits of antioxidants are now recognized to be predominantly dependent on their ability to reach specific sites, making mitochondrial-targeted delivery a primary research objective in ongoing research. Moreover, several studies have shown curcumin’s therapeutic potential in various diseases, though few studies exhibit consistent results on optimal curcumin concentration for promoting oocyte maturation. Additionally, insufficient studies exist on curcumin’s long-term effects on oocyte developmental competence. Therefore, future research is needed to focus on testing the proposed solutions and their related effects on follicle growth and development to treat infertility, along with clinical trials to explore the mechanisms behind curcumin’s bioactivity.

## Figures and Tables

**Table 1:** Modulatory effects of curcumin on ovarian follicular development (indicated by alterations in oxidative stress and antioxidant biomarkers): A comparative insight from *in vivo* and *in vitro* studies.

In vivo/ In vitro	Experimental Animal	Study Period	Pro- Oxidative Stimulus	Curcumin (CUR) Dose	Observations	Increase (↑) and Decrease (↓)	Reference
In vivo	Wister Rats (8 weeks old)	14 d	Intraperitoneal injection of 10 mg/kg formaldehyde (FA) for 14 days	FA + CUR (10 mg/kg/d + 100 mg/kg/d)	Follicular destruction and atresia	↓	[106]
					Ovarian follicle count	↑	
					GPx protein levels	↑	
					FIGLA levels	↑	
					Apoptosis rate	↓	
					TAC/TOS ratio	↑	
In vivo	Balb/c Mice (6–8 weeks old)	35 d	Intragastrical injection of 50 mg/kg of Acrylamide (ACR) for 35 days	ACR + CUR (50 mg/kg/d + 100 mg/kg/d) and ACR + CUR (50 mg/kg/d + 200 mg/kg/d)	SOD, CAT and GPx protein levels	↑	[119]
					Bax and Caspase-3	↓	
					Bcl-2 levels	↑	
In vivo	Kunming Mice (6–8 weeks old)	16 d	Intraperitoneal injection of 8 mg/kg of Sodium arsenite (As) for 16 days	Curcumin dose-dependent groups were as follows: 0 mg/kg/d, 100 mg/kg/d, 150 mg/kg/d and 200 mg/kg/d	SOD and GPx	↑	[107]
					MDA levels	↓	
					P66Shc expression levels	↓	
					Proliferation of granulosa cells	↑	
In vivo	Wister Albino Rats (3–4 months old)	14 d	Intraperitoneal injection of 200 mg/kg of Cyclophosphamide (POF) on day 1 and then 8 mg/kg for 14 days	POF + CUR (8 mg/kg/d + 100 mg/kg/d)	ROS levels (SOD, CAT, GPx levels ↑)	↓	[111]
					GSH levels and estradiol levels	↑	
					FSH and LH levels	↓	
					KF-kB levels	↑	
					MAPK levels	↓	
					Bax and Caspase-3	↓	
In vivo	C5BL/6 Mice (7–8 weeks old)	42 d	Subcutaneous Injection of 200 mg/kg D-galactose for 24 days	D-gal + CUR (200 mg/kg/d + 100 mg/kg/d)	SOD activity	↑	[105]
					MDA activity	↓	
					Amh expression level	↑	
					Caspase-3 and -9 protein expression levels	↓	
					E2 levels	↑	
					FSH and LH levels	↓	
					Curcumin inhibited ovarian injury via the Nrf2/HO-1 and P13K/Akt pathways		
In vivo	ICR Mice (8 weeks)		Oral administration of 0.75 mg/kg of aflatoxin B1 (AFB1) for 28 days	AFB1 + CUR (0.75 mg/kg/d + 100 mg/kg/d)	Curcumin restored normal follicular structure		[120]
					Atretic follicles	↓	
					ROS and MDA levels	↓	
					PI3K/Akt expression levels	↑	
					Expression of antioxidant markers (including SOD, GPx, NF-kB, Nrf2, HO-1 and HSP70)	↑	
					Bax and Caspase-3	↓	
In vitro	Porcine Granulosa Cells	24 h	Aflatoxin B1 (AFB1) at a concentration of 8 µM for 12 h.	Groups: Control, AFB1 (8 µM), CUR (10 µM) and AFB1+ CUR (8 µM + 10 µM)	Curcumin preserved the AFB1-induced inhibition of cell viability, mitochondrial dysfunction, oxidative stress, cell cycle arrest and apoptosis		
					ROS and MDA levels	↓	
					SOD and GPx levels	↑	
					PI3K/Akt expression levels	↑	
					Mitochondrial fusion genes (Mfn1 and Mfn2)	↑	
					Mitochondrial fission genes (Drp1 and Fis1)	↓	
					Bcl-2 and XIAP	↑	
					Expression of apoptosis markers (Bax, BAD, Casepase-3, PTEN and AIF)	↓	
In vivo	ICR Mice (6–8 weeks old)	14 d	Intraperitoneal injection of 20 mg/kg/d of 3-nitropropionic acid (3-NPA for 14 days	Curcumin dose-dependent groups were as follows: 50 mg/kg/d, 100 mg/kg/d and 200 mg/kg/d	No. of primary and secondary follicles	↑	[121]
					Atretic follicles	↓	
					ROS and MDA levels	↓	
					Bcl-2 and SOD expression levels in 100 mg/kg/d and 200 mg/kg curcumin-treated groups	↑	
					Bax and Cl-cas3 expression levels	↓	
					LC3B2 and BECNI levels	↓	
					PAMK/AMAK levels	↓	
					pmTOR/mTOR expression levels	↑	
In vitro	ICR Mice (6–8 weeks old)	24 h	H_2_O_2_ at a concentration of 50 µM, 100 µM, 200 µM and 400 µM for 3 h respectively in groups	Three curcumin dose-dependent groups were as follows: 5 µM, 10 µM and 20 µM	Cell viability	↓	
					Bcl-2 expression levels	↑	
					Bax and Cl-cas3 (dose dependently)	↓	
					LC3B2 and BECN1 expression levels	↑	

## Data Availability

Data are contained within the article.
